# Chronic widespread dermatophytosis due to *Trichophyton rubrum*: a syndrome associated with a *Trichophyton*-specific functional defect of phagocytes

**DOI:** 10.3389/fmicb.2015.00801

**Published:** 2015-08-04

**Authors:** Maria da Glória T. de Sousa, Grazielle B. Santana, Paulo R. Criado, Gil Benard

**Affiliations:** ^1^Laboratory of Medical Investigation Unit 53, Division of Clinical Dermatology, Medical School, University of São PauloSão Paulo, Brazil; ^2^Laboratory of Medical Mycology, Tropical Medicine Institute, University of São PauloSão Paulo, Brazil; ^3^Laboratory of Medical Investigation Unit 56, Division of Clinical Dermatology, Medical School, University of São PauloSão Paulo, Brazil; ^4^Division of Clinical Dermatology, Clinics Hospital, Medical School of the University of São PauloSão Paulo, Brazil

**Keywords:** innate immunity, immunodeficiency, dermatophytosis, macrophages, neutrophils, *Trichophyton rubrum*

## Abstract

Dermatophytes are agents of typically benign superficial infections. However, an increasing number of severe infections in immunocompromised hosts has been reported. We aimed to understand the factors underlying the existence of a cohort of patients presenting with chronic widespread dermatophytosis (CWD) due to *Trichophyton rubrum*, but with no signs of immunodeficiency. Their disease is usually recurrent and difficult to manage. Fourteen patients meeting the following criteria for CWD were studied: *T. rubrum* culture-proven skin lesions of ≥10 cm in at least one dimension; the involvement of at least three non-contiguous localizations of >1 year’s duration; and no predisposing conditions. For comparison, we also studied 13 acute *Tinea pedis* patients. Macrophages and neutrophils were isolated and tested for *T. rubrum* conidia phagocytic and killing activity. H_2_O_2_, NO, and pro- and anti-inflammatory cytokine release were measured. All experiments were run with age- and sex-matched healthy donors’ cells in parallel. CWD patients’ macrophages and neutrophils presented with reduced *T. rubrum*–phagocytic and killing abilities, and reduced H_2_O_2_ and NO release when compared with those of healthy donors. CWD patients’ macrophages secreted lower levels of the proinflammatory cytokines interleukin (IL)-1β, IL-6, IL-8, and tumor necrosis factor (TNF)-α, but enhanced levels of the anti-inflammatory cytokine IL-10. Neutrophil secretion closely followed this unbalanced pattern. In contrast, responses to the positive controls zymosan, lipopolysaccharide, and phorbol myristate acetate were comparable with those of healthy donors. The same experiments were performed with macrophages and neutrophils from the acute *Tinea pedis* patients and showed no differences when compared with the matched healthy donors. Patients with CWD have a *T. rubrum*-related functional deficiency of phagocytes and may represent a distinct clinical entity in the complex spectrum of the *Trichophyton*–host interaction.

## Introduction

Dermatophytes are agents of typically benign superficial infections, of which the non-inflammatory, scaly lesions of toe webs due to *Trichophyton rubrum* are known as the most common example (Seebacher et al., 2008). However, there are numerous reports of severe and occasionally life-threatening dermatophytic infections in the increasing population of immunocompromised patients, showing that dermatophytoses may pose a more serious threat to these patients (Marconi et al., 2010). Recently, the immune–genetic background underlying deep (invasive) dermatophytic infections aﬄicting some members of consanguineous families in Northern Africa has been elucidated (Lanternier et al., 2013). This syndrome was associated with an autosomal recessive CARD9 deficiency that accounts for the patients’ failure to limit the invasiveness of dermatophytes such as *T. rubrum* and *T. verrucosum*. CARD9 is an adaptor protein downstream to several immune receptors such as dectin-1, dectin-2, and mincle, which recognize fungal structures and are critical for antifungal Th-17 responses (Lionakis and Holland, 2013). On the other hand, there are also frequent reports of patients presenting with recurrent or chronic widespread dermatophytosis (CWD) of the skin which, although not invasive, is difficult to manage (Sentamilselvi et al., 1997–1998; Vittorio, 1997; Gorani et al., 2002; Cordeiro et al., 2006; Seyfarth et al., 2007; Balci and Cetin, 2008; Kong et al., 2015).

Patients with acute superficial dermatophytosis are able to mount cell-mediated immune (CMI) responses against the causative agent, which has been associated with resolution of the infection (Hanifin et al., 1974; Hay and Shennan, 1982). In contrast, those who suffer from chronic or recurrent infections are unable to develop a CMI response (Hanifin et al., 1974; Hay and Shennan, 1982). The reasons for this inability are not yet known. Here, we describe a series of dermatophytosis patients with a distinct clinical presentation, chronic, or recurrent widespread involvement, in whom we detected a deficiency of phagocytes (macrophages and neutrophils) to handle its apparently single causative agent, *T. rubrum*.

## Materials and Methods

### Patients and Controls

From January 2013 to March 2014, patients with CWD and no known predisposing conditions referred to our admission service were invited to participate in the study. Written informed consent was obtained from all patients prior to blood collection, and the study was approved by the Human Experimentation Ethics Committee of the Hospital das Clínicas, Universidade de São Paulo in Brazil (#0837/10). Patients were diagnosed with CWD if they had *T. rubrum* culture-proven dermatophytosis involving at least three non-contiguous localizations, with lesions (typically well-delimited plaques) of ≥10 cm in at least one of its extensions, for more than 1 year. Onychomycosis was not considered in the criteria. Bilateral involvement of the feet was considered as a single localization. Patients should not have presented with any condition that could potentially interfere with their immune system (pregnancy, immune-mediated, or inflammatory conditions, infectious diseases [including HIV and hepatitis C virus], diabetes mellitus, Cushing’s syndrome, alcoholism, and topical or systemic treatments featuring immunosuppressive drugs). No patients had any potential occupational/professional risk for dermatophytosis, such as prolonged contact with water, working in warm/humid environments, or the use of special clothes. A second part of the study involved the recruitment of individuals presenting with *Tinea pedis* (*Tp*), the most common and benign form of dermatophytosis due to *T. rubrum* in Brazil (Costa-Orlandi et al., 2012). From March to June 2014, a total of 13 patients with *Tp* were enrolled using the same exclusion criteria as described above for the CWD patients.

For both studies, healthy donors that were age- (±3 years) and sex-matched with the CWD and *Tp* patients served as controls. All experiments were run with the matched control’s cells in parallel.

### *Trichophyton rubrum* Conidia Preparation

*Trichophyton rubrum* ATCC28188 was streaked onto potato dextrose agar plates to isolate individual colonies for 12 days. Colonies were cultured in a shaking incubator for 72 h at 30°C in potato broth for the *in vitro* assays. The conidia were filtered to remove hyphae and washed with phosphate buffered saline (PBS) before use. For fluorescence labeling, washed conidia were labeled with carboxyfluorescein succinimidyl ester (CFSE, 100 μg/mL; Life Technologies, Eugene, OR, USA) for 30 min at 25°C, followed by extensive washing.

### Macrophage and Neutrophil Interaction with *T. rubrum* Conidia

Human monocyte-derived macrophages and human neutrophils were obtained from peripheral blood mononuclear cell (PBMC) leukocytes, as described previously (Böyum, 1968; Calvi et al., 2003). Specifically, for the generation of macrophages, human PBMCs were isolated by centrifugation over a Ficoll–Paque^TM^ PLUS (GE Healthcare Bio-Sciences Corp., Piscataway, NJ, USA) gradient. Monocytes were purified by adherence on gelatin-coated plates for 1 h, followed by extensive washing to remove non-adherent cells. After at least 12 h of incubation, monocytes (day 1) were harvested and then differentiated into day 4 and day 7 macrophages via culturing in Roswell Park Memorial Institute (RPMI) medium with 10% fetal calf serum (FCS). Viability was >95%, as determined by trypan blue dye exclusion. The macrophages were then plated the night before use, while neutrophils were plated on the same day in 24-well plates at a density of 3 × 10^5^ cells/well in RPMI–10% heat-inactivated FCS. For neutrophil isolation, following centrifugation over Ficoll–Paque, the granulocytes were isolated from the bottom part of the tube containing red cells, using dextran and saline as previously described (Böyum, 1968). This yielded neutrophils with >93% purity and ≥90% viability by trypan blue dye exclusion. For the *in vitro* binding and cytokine assays, unlabeled or CFSE-labeled *T. rubrum* conidia were added to the cells, as indicated, and incubated for 30 min at 37°C. In some experiments, unlabeled or fluorescein isothiocyanate-labeled zymosan (25 particles/cell; Thermo Fisher Scientific) and mannan (1 mg/mL; Sigma–Aldrich Co., St Louis, MO, USA) were added alone as indicated. Unbound particles were removed by washing. The medium was replaced, and the cells were cultured for either further 3 h at 37°C and 5% CO_2_ for the analysis of tumor necrosis factor (TNF)-α or 18 h for the analysis of the other cytokines. After the 3-h incubation period, supernatants were stored at –80°C until cytokine determination, while the cells were lysed in 3% (volume/volume) Triton^®^ X-100, and the cell-associated fluorescence was measured as the mean florescence intensity (Filtermax-F5; Molecular Devices LLC, Sunnyvale, CA USA). Cytokine release was not influenced by the presence of the fluorescent label on the fungal particles (not shown). Lipopolysaccharide (LPS; Sigma–Aldrich Co.) was used as the positive control for cytokine release. For the killing assays, macrophage and neutrophils (3 × 10^4^ cells) were co-cultured with non-opsonized *T. rubrum* conidia at a 5:1 ratio in 96-well plates for 3 h at 37°C and 5% CO_2_. The phagocytes were then washed to remove unbound conidia and lysed in Triton^®^ X-100, as previously described (Horwitz and Silverstein, 1980). The supernatants’ serial dilutions were plated in duplicate in Petri dishes with 15 mL of Sabouraud agar and incubated for 5 days at 28°C. Colonies were counted manually.

### H_2_O_2_ Measurement

H_2_O_2_ release by phagocytes was measured with the horseradish peroxidase–phenol red oxidation method described previously (Calvi et al., 2003). Briefly, phagocytes co-cultured with *T. rubrum* conidia or stimulated with 100 nM of phorbol-12-myristate-13-acetate (PMA; Sigma–Aldrich Co.) were centrifuged at 200 *g* for 10 min at 4°C and were re-suspended in 1 mL of phenol red buffer containing 50 μg/mL of horseradish peroxidase type II (Sigma–Aldrich Co.). Aliquots of 100 μL were then transferred to 96-well culture plates and were incubated for 1 h at 37°C and 5% CO_2_. The reaction was stopped by the addition of 10 μL of 1N NaOH, and the absorbance was read at 620 nm (Micro-ELISA reader, Filtermax-F5). The absorbance was transformed into a standard curve of H_2_O_2_ that was serially diluted from 3.2 to 200.0 μM H_2_O_2_/mL.

### NO Measurement

Supernatants were diluted 1:10 in deionized water and NO measurement was performed through chemiluminescence reaction between ozone and NO generated by the reduction of the sample with vanadium chloride in acid at 95°C, using a Nitric Oxide Analyzer Model 208A (Sievers Instruments Inc., Boulder, CO, USA) as described (Alves et al., 2002). The assay was standardized by a calibration curve of nitrate (0.05–20.0 μM/mL) obtained from sodium nitrate.

### Cytokine Measurements

Supernatants harvested from phagocyte–*T. rubrum* co-cultures were measured for interleukin (IL)-1β, IL-6, IL-8, IL-10, IL-12p70, and TNF-α using a cytometric bead array (BD, Franklin Lakes, NJ, USA). Detection limits were, respectively, 7.2, 2.5, 3.6, 3.3, 1.9, and 3.7 pg/mL.

### Statistical Analysis

The Wilcoxon signed-rank test was used to compare the two patient groups (CWD and *Tp*) with the respective paired control groups (GraphPad Prisma; GraphPad Software, Inc., La Jolla, CA, USA). Statistical significance was set at *P* < 0.05.

## Results

### Patients’ Clinical Characteristics

**FIGURE 1:**
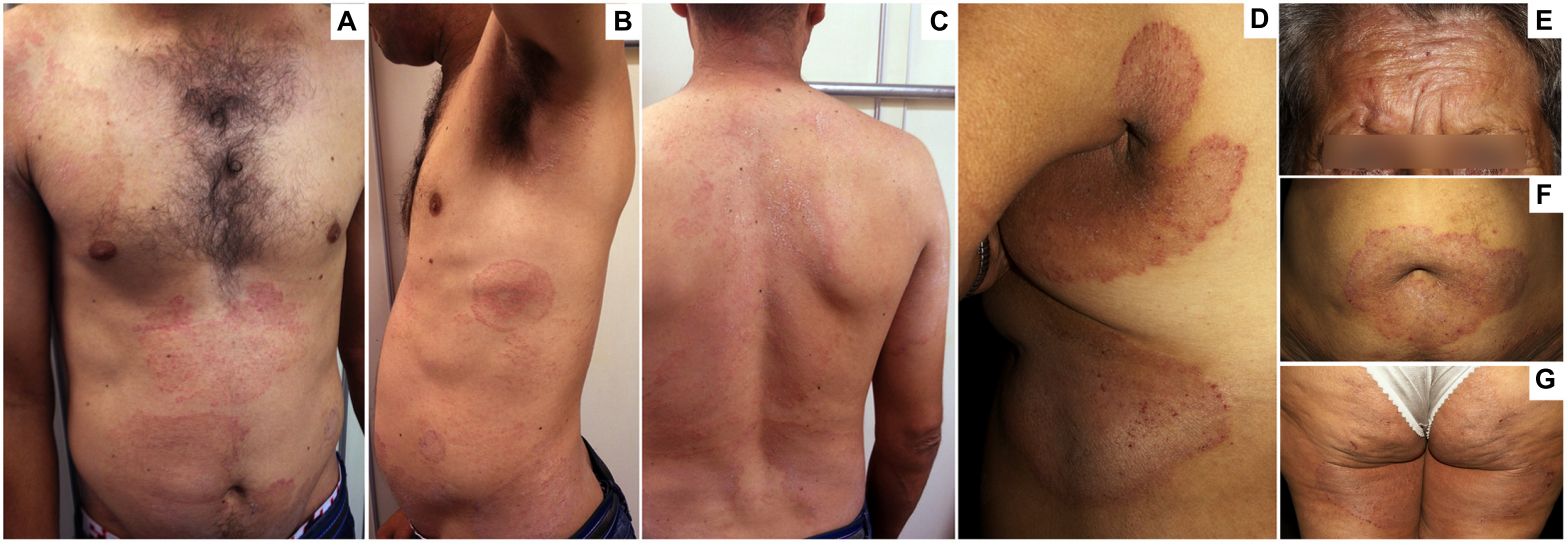
**Clinical aspects of chronic widespread dermatophytosis (CWD) due to *Trichophyton rubrum*. (A–C)** Several well-delimited, scaly, and slightly erythematous plaques in the abdomen **(A–C)**, thorax **(A,B)**, shoulders **(A)**, arms **(B)**, neck **(C)**, and back **(C)** in a 36-year-old man with a 4-year history of dermatophytosis. **(D–G)** Well-delimited, scaly, and erythematous plaques in the face **(E)**, the periumbilical **(F)**, and submammary **(D)** regions; as well as the armpits **(D)**, thighs **(D)**, and buttocks **(G)** in a 60-year-old woman with a 5-year history of dermatophytosis.

A total of 14 patients with CWD of the skin were diagnosed (10 males and 4 females). There were two Afro-Brazilians, one Asian, and the remaining patients were of mixed ethnic origin. None of the patients were from consanguineous families. The patients’ age range was 22–61 years (mean: 46 years). Lesions were present for >3 years in all patients (range: 3–16 years), except for one patient with 1 year of lesions. As shown in **Figure 1**, patients had typical erythematous, slightly elevated, scaly, and well-delimited lesions over different, non-contiguous body segments, particularly those of glabrous skin. Small satellite lesions were also frequent. The distribution of the lesions was as follows: feet (bilateral) in nine patients; hands (usually bilateral) in seven patients; crural in seven patients; legs in six patients; trunk in six patients; buttocks in five patients; arms in three patients; axillas in three patients; neck in two patients; and ears in two patients. In addition, ten patients had concomitant onychomycosis. A history of atopy (asthma) was evident in only two patients. With the exception of the youngest patient, who had a twin brother with chronic *T. rubrum* lesions (who refused to participate in the study), no other patients reported having close relatives with similar manifestations. All patients reported previous antifungal treatments, either topical or systemic, some of which included terbinafine; compliance to the treatments, however, could not be assessed. Four patients reported apparent resolution with relapse after some months, while seven reported no or only partial improvement and three provided no reliable information. No other fungi (*Candida* sp. and dermatophytes) were isolated from the lesions. CWD patients’ laboratory screening was within normal limits, including glycemic levels, hemoglobin levels, white blood cell counts, and liver and renal function tests.

### Phagocytic and Killing Abilities against *T. rubrum* of Macrophages and Neutrophils from CWD Patients

**FIGURE 2:**
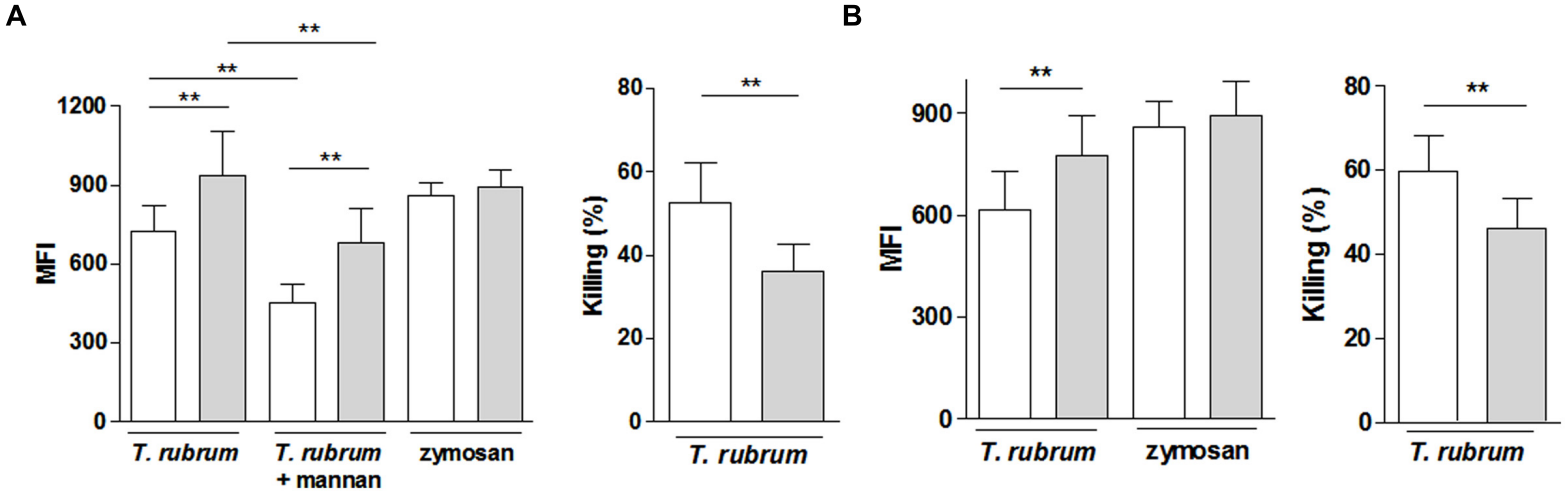
**Reactivity of phagocytes (**A**, macrophages; **B**, neutrophils) from patients with CWD (clear bars) and age- and sex-matched healthy controls (gray bars) to *T. rubrum***. Phagocytosis of CFSE-labeled *T. rubrum* conidia was assessed by fluorometry as the mean florescence intensity (MFI). Killing activity is shown as the % of the CFU counts in the control wells (conidia suspension in absence of phagocytes). Phagocytosis, *n* = 14; microbicidal activity, *n* = 10. Results are shown as the mean ± SEM. **P < 0.01.

Macrophages from CWD patients presented with a slight but significantly lower ability to phagocytize *T. rubrum* compared with age- and sex-matched healthy controls, while the ability to phagocytize zymosan particles was similar (**Figure 2A**). *T. rubrum* phagocytosis was significantly blocked by mannan in both groups. In presence of the polysaccharide, phagocytosis by CWD macrophages remained lower than in controls. The lower phagocytic ability was reflected by the lower killing ability of CWD macrophages (**Figure 2A**). Experiments conducted in parallel showed the same modest but significant deficiency in the phagocytic and killing abilities of the CWD patients’ neutrophils (**Figure 2B**).

### Release of the Cytotoxic Molecules H_2_O_2_ and NO by Macrophages and Neutrophils Challenged with *T. rubrum*

**FIGURE 3:**
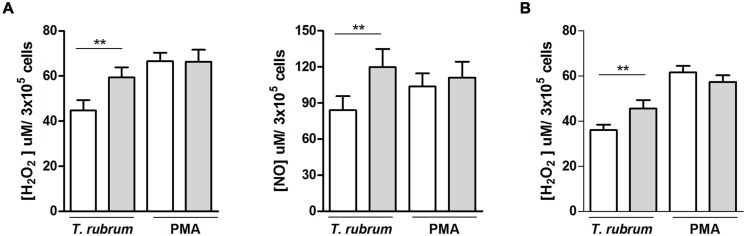
**NO and H_2_O_2_ release by phagocytes (**A**, macrophages; **B**, neutrophils) from patients with CWD (clear bars) and age- and sex-matched healthy controls (gray bars) in the presence of *T. rubrum* or PMA.** NO and H_2_O_2_ release in culture supernatants were measured by fluorometric assays. H_2_O_2_, *n* = 14; NO, *n* = 9. Results are shown as the mean ± SEM. **P < 0.01.

Chronic widespread dermatophytosis patients’ macrophages released slightly but significantly less H_2_O_2_ and NO in the presence of *T. rubrum* than those of the control individuals (**Figure 3A**). Again, the non-specific ability to release these molecules was intact in the patients’ macrophages, as shown by their preserved response to PMA. H_2_O_2_ release by CWD patients’ neutrophils showed similar results: there was a decrease in the presence of *T. rubrum*, but there was also a preserved response to PMA (**Figure 3B**).

### Cytokine Secretion by Macrophages and Neutrophils Challenged with *T. rubrum*

**FIGURE 4:**
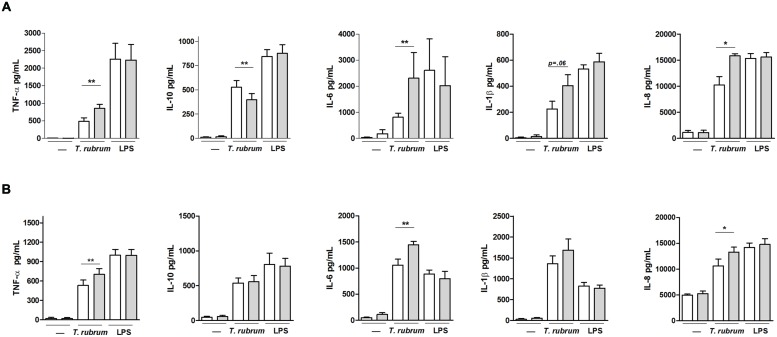
**Cytokine levels in the supernatants of **(A)** macrophages and **(B)** neutrophils from patients with CWD (*n* = 8, clear bars) and age- and sex-matched healthy controls (gray bars) cultured in the presence of *T. rubrum* conidia, LPS, or medium alone (–).** Supernatants were collected after 3 h (TNF-α) and 18 h (IL-1β, IL-6, IL-8, and IL-10), and cytokine levels were measured by CBA. Results are shown as the mean ± SEM. *P < 0.05; **P < 0.01.

The supernatants of macrophages and neutrophil co-cultures with *T. rubrum* were collected and evaluated for proinflammatory (IL-1β, IL-6, IL-8, IL-12p70, and TNF-α) and anti-inflammatory (IL-10) cytokines. With the exception of IL-12p70, where its levels were always below the detection limit (1 pg/mL), both macrophages and neutrophils secreted substantial levels of these cytokines (**Figure 4**). However, consistent with the lesser anti-*T. rubrum* activity of CWD patients, the amounts of proinflammatory cytokines released by CWD macrophages upon *T. rubrum* challenge were significantly lower than those of the matched controls, whereas IL-10 release was significantly increased. On the other hand, the amounts elicited in the positive control wells (LPS) and in the non-stimulated wells (medium only) were similar between CWD patients and controls for every cytokine. Neutrophils followed a similar pattern: there was reduced release of the proinflammatory cytokines upon *T. rubrum* challenge (except for IL-1α, which reduction did not reach statistical significance, and IL-10, that was not increased), and no differences between patients and controls with PMA or medium alone were noted.

### Macrophage and Neutrophil Responses of Acute *Tp* Patients

Subsequently, the same series of experiments were performed with phagocytes isolated from *Tp* patients and age- and sex-matched healthy controls. Results of these experiments are shown in Supplementary Tables S1 (macrophage responses) and S2 (neutrophil responses). Both macrophages and neutrophils from *Tp* patients exhibited a similar ability to phagocytize, kill, and release H_2_O_2_, NO, and cytokines (IL-1β, IL-6, IL-8, IL-10, and TNF-α) upon challenge with *T. rubrum* as controls. There were also no differences between the patients and controls’ responses in the positive control wells (zymosan, LPS, or PMA).

## Discussion

We were interested in understanding the factors underlying the existence of a distinct cohort of patients presenting with longstanding and widespread dermatophytosis due to *T. rubrum*, but with no other clinical evidence of immunodeficiency. Remarkably, their disease was refractory to apparently appropriate treatments. Here, we showed that macrophages and neutrophils from these patients presented with reduced phagocytic and killing abilities, as well as with disturbed immunoregulatory properties (cytokine secretion), upon challenge with *T. rubrum* conidia. These findings may help explain why some individuals develop recalcitrant and widespread disease due to a fungus that typically causes a localized and easily manageable superficial infection. Of note is that despite the chronicity and extension of the skin involvement, in all patients it exhibited little inflammation and no evidence of invasion of deeper tissues.

Earlier studies reported that resolution of an acute episode of a dermatophyte infection was associated with the development of a specific CMI response that protected, at least partially, against reinfections (Jones et al., 1973). Further *in vitro* and *in vivo* studies demonstrated that these responses were predominantly Th-1 mediated (Miyata et al., 1996; Koga et al., 2001; Brasch, 2009; Santiago et al., 2014), although recent evidence in experimental models of dermatophytosis also pointed to Th-17 responses (Cambier et al., 2014). Chronic infections, on the other hand, were associated with a lack of CMI responses (Hanifin et al., 1974; Hay and Shennan, 1982). Importantly, there are few and conflicting data regarding predisposing genetic traits in chronic *Tp* (Svejgaard et al., 1983; Ahmed et al., 1985). Although patients with CWD are frequently described in the literature (Sentamilselvi et al., 1997–1998; Vittorio, 1997; Gorani et al., 2002; Cordeiro et al., 2006; Seyfarth et al., 2007; Balci and Cetin, 2008; Kong et al., 2015), it is not yet known why they are unable to mount CMI responses and resolve the infection. Our present findings may provide some clues to this issue. Both neutrophils and macrophages are actively attracted to the site of acute *T. rubrum* infection in patients and experimental models of dermatophytosis (Hay et al., 1988; Brasch and Sterry, 1992; Brasch, 2009), although only neutrophils penetrate the epidermis and interact with *T. rubrum* (Hay et al., 1988). Monocyte-derived macrophages are an important cellular component in the dermis inflammatory infiltrate of dermatophytosis (Hay et al., 1988; Brasch and Sterry, 1992), thus playing a role in the induction (or lack thereof) of effective CMI responses. The main antigen-presenting cell (APC) in the epidermis is the Langerhans cell (LC), which increases in number in the dermatophyte-infected epidermis and also interacts with *T. rubrum* (Brasch et al., 1993). As the study of patients’ epidermal LCs was not possible, we focused on their precursors, peripheral blood monocytes (Ginhoux et al., 2006). Both human neutrophils and monocyte-derived macrophages are normally able to phagocytize and kill *T. rubrum* (Calderon, 1989). The participation of reactive oxygen species in this killing has been demonstrated (Calderon, 1989). NO also exhibits toxic activity against a range of fungi, including *T. rubrum* (Regev-Shoshani et al., 2013). In our chronic CWD patients, both phagocytes presented with reduced rates of phagocytosis and killing activity when challenged with *T. rubrum*, as compared with the age- and sex-matched controls. The macrophages’ phagocytosis was partially blocked by mannan, which was expected since mannose receptors are important in the recognition of fungi harboring mannan in their cell walls; however, even after blockade, the phagocytosis index was lower in CWD patients than in controls. These findings are consistent with the reduced peroxide and NO release by both CWD patients’ neutrophils and macrophages. It is important to note that the functional impairments in the presence of *T. rubrum* were frequently partial, with values around 20–30% below those of the matched healthy individuals, while the responses to the positive control stimuli (zymosan particles or PMA) were fully preserved. This suggests a partial but specific deficiency, consistent with the limited clinical severity of the disease (e.g., the absence of invasion) and the lack of susceptibility to other fungi (e.g., *Candida* sp.); however, it was apparently strong enough to slow the elimination of the fungus from the epidermis and hinder the capture of antigens for subsequent processing, presentation, and elicitation of the CMI response, which is in agreement with the chronic and widespread behavior of the infection.

This mechanism is reinforced by the altered pattern of cytokines released by the CWD patients’ phagocytes. Macrophages secreted lower levels of the proinflammatory cytokines IL-1β, IL-6, IL-8, and TNF-α, although they enhanced levels of the anti-inflammatory cytokine IL-10 in response to *T. rubrum* but not to LPS. Neutrophils’ secretion closely followed this unbalanced pattern. IL-1β is a key cytokine that drives a proinflammatory state and instructs adaptive immune responses (Dinarello, 2009); this latter function was due to the cytokine’s ability to induce the differentiation of monocytes into macrophages with both enhanced phagocytic and antigen-presenting functions (Schenk et al., 2014). In addition, IL-1β has recently been shown to be a key mediator in the IFN-γ-induced control of *T. rubrum* proliferation in an experimental model of dermatophytosis (Baltazar et al., 2014). In addition to its systemic proinflammatory activity, IL-6 is engaged along with IL-1β in the establishment of Th-17 responses (Dinarello, 2009). Furthermore, IL-8 and TNF-α are also potent inducers of systemic inflammation and chemoattractants to neutrophils; in addition, TNF-α is crucial for APC activity (de Luca and Gommerman, 2012). Conversely, IL-10 has the ability to counteract most of these proinflammatory activities. Thus, the reduced levels of the proinflammatory cytokines with Th-1/Th-17-inducing properties, associated with the enhanced IL-10 release by macrophages, likely adversely affected the induction of an effective CMI response.

Two additional points from the present work deserve to be stressed. First, the observed immunological abnormalities were specific to patients with CWD, in the sense that the same experiments were carried out with the cells of patients with *Tp* alone; these experiments showed no differences when compared with the age- and sex-matched healthy controls. Second, it is possible that the reduced killing activity of the CWD phagocytes could be related to the impaired phagocytosis. Although the phagocytes’ killing mechanisms of *T. rubrum* are not well defined, in many pathogenic fungi phagocytosis and killing are only partially related events as killing mechanisms may also occur in the extracellular milieu through the release of stressing molecules (Amulic et al., 2012). Moreover, as shown by Campos et al. (2006) in an *in vitro* mouse model of *T. rubrum* infection, the ingested conidia could survive and elongate inside the phagocytes, leading to the death of these cells (Campos et al., 2006). Thus, *T. rubrum* killing by phagocytes would eventually depend more on the release of extracellular toxic components, an important point that warrants further studies.

Collectively, these immunological data strongly support the widespread and recurrent nature of this infection, as illustrated in **Figure 1**. Although some authors defined *Tp* as the primary site of lesions and the reservoir in chronic *T. rubrum* infections (based on local epithelial features that would hinder eradication of the fungi; Zaias and Rebell, 1996), six of our 15 patients lacked *Tp*. Thus, concomitant chronic *Tp* infection, although frequent, does not seem to be a prerequisite for CWD. Also, a “chronic trichophytosis syndrome” has been proposed in Croatian patients due to *T. mentagrophytes var. interdigitalis*, which predominantly involves the feet (Gregurek-Novak et al., 1993). As in our patients, this chronic trichophytosis syndrome was associated with decreased leukocyte phagocytosis and digestion, although in this case, the defect was non-specific, as it was demonstrated using only sheep erythrocytes as a target.

Regularly viewed as a well-adapted and low-virulence pathogen (Dahl and Grando, 1994), the *T. rubrum* human–host interaction appears to be rather complex, giving rise to a spectrum of diseases depending on the quality of the host’s defenses. This spectrum ranges from the benign and localized *tinea* on the one hand, where the host’s defenses appear to be intact, to the life-threatening systemic infections on the other, as seen in CARD9-deficient (Lanternier et al., 2013) or severely immunocompromised patients [e.g., cirrhotic patients (Marconi et al., 2010)]. Other clinical entities would stand in between, such as the deep infections in immunocompromised patients (AIDS, hematological malignancies, and solid organ transplant recipients), who are still able to avoid systemic spread, possibly owing to preserved innate immune responses (Tsang et al., 1996; Franco, 2001; Smith et al., 2001). We propose that our series of patients could represent a distinct clinical entity – namely, chronic or recurrent widespread dermatophytosis – secondary to a subtle and apparently *Trichophyton*-specific innate immune deficiency, which also stands in between the two poles.

Chronic widespread dermatophytosis needs further characterization, such as by identifying the molecular pathways underlying its phagocyte dysfunction and its possible genetic traits. An association with mutations in the *CARD-9* gene seems unlikely due to the lack of familial segregation in all but one of the 14 patients, as well as to the less severe, non-invasive behavior of the CWD. Another interesting aspect would be to study the patients’ epithelial LCs. Regarding the patients’ response to treatment, preliminary results of the follow up of our CWD patients indicated that only half of them showed an initial good response to terbinafine; they are now being followed for recurrences. The other half of our patients persisted with residual *T. rubrum*-positive lesions (Santana GB, Bezerra TA, and Moraes-Vasconcelos D, unpublished data).

## Conclusion

As for the insights provided by the study of the immune mechanisms underlying the different outcomes of the host–*Candida* interaction (Lionakis and Holland, 2013), the study of the also rather complex *Trichophyton*–host interaction may uncover additional mechanisms associated with either protection or susceptibility to fungal infections.

## Conflict of Interest Statement

The authors declare that the research was conducted in the absence of any commercial or financial relationships that could be construed as a potential conflict of interest.
