# Supplementary material for: Chronic widespread dermatophytosis due to Trichophyton rubrum: a syndrome associated with a Trichophyton-specific functional defect of phagocytes
Source: Front Microbiol. 2015 Aug 4;6:801. doi: 10.3389/fmicb.2015.00801 (PMC4523820; doi:10.3389/fmicb.2015.00801)
Supplement: Supplementary file 1 [file Table_1.PDF]

Supplementary Table 1. Comparison of macrophages responses to *T. rubrum* and positive control stimuli between *Tinea pedis* patients (Tp) and sex and age-matched healthy donors

	Phagocytosis of *T. rubrum* (n=13)	H_2O_2 release µM/3 x 10^5 cells (n=8)		NO release µM/ 3x10^5 cells (n=9)		TNF secretion (pg/mL) (n=6)			IL-10 secretion (pg/mL) (n=6)			IL-6 secretion (pg/mL) (n=6)			IL-1β secretion (pg/mL) (n=6)			IL-8 secretion (pg/mL) (n=6)		
		Tr	PMA	Tr	PMA	Bg	Tr	LPS	Bg	Tr	LPS	Bg	Tr	LPS	Bg	Tr	LPS	Bg	Tr	LPS
Tp	684 (327)	41 (4)	60 (5)	99 (15)	101 (15)	36 (13)	548 (93)	1918 (499)	4 (2)	389 (60)	1918 (499)	46 (10)	890 (134)	963 (185)	19 (10)	714 (79)	837 (57)	46 (10)	890 (134)	963 (185)
Cont	740 (312)	43 (3)	63 (4)	111 (16)	202 (12)	35 (13)	586 (51)	1517 (422)	24 (11)	420 (43)	487 (162)	100 (52)	789 (126)	991 (223)	13 (4)	729 (130)	786 (57)	100 (52)	789 (126)	991 (223)
P=	0,15	0,44	0,57	0,36	0,73	0,8	0,69	0,22	0,13	0,3	1,00	0,31	0,63	0,84	1,0	0,81	0,31	0,22	0,22	0,44

Tr, *Trichophytom rubrum* conidia; PMA, phorbol 12-myristate 13-acetate; LPS, lypopolyssacharide, Bg, background or without stimulation; Cont, healthy donors

Data presented as mean (SE)
