# Supplementary material for: Chronic widespread dermatophytosis due to Trichophyton rubrum: a syndrome associated with a Trichophyton-specific functional defect of phagocytes
Source: Front Microbiol. 2015 Aug 4;6:801. doi: 10.3389/fmicb.2015.00801 (PMC4523820; doi:10.3389/fmicb.2015.00801)
Supplement: Supplementary file 2 [file Table_2.PDF]

Supplementary Table 2. Comparison of neutrophils responses to *T. rubrum* and positive control stimuli between *Tinea pedis* patients (Tp) and sex and age-matched healthy donors

	Phagocytosis of *T. rubrum* (n=13)	H_2O_2 release µM/3 x 10^5 cells (n=8)		TNF secretion (pg/mL) (n=8)			IL-10 secretion (pg/ml) (n=8)			IL-6 secretion (pg/ml) (n=6)			IL-1β secretion (pg/mL) (n=6)			IL-8 secretion (pg/mL) (n=6)		
		rubrum	PMA	Bg	Tr	LPS	Bg	Tr	LPS	Bg	Tr	LPS	Bg	Tr	LPS	Bg	Tr	LPS
Tp	559	45	63	28	406	960	14	366	579	11	712	722	11	630	1247	3560	9338	10991
	(91)	(4)	(6)	(12)	(73)	(119)	(8)	(61)	(109)	(9)	(153)	(85)	(9)	(83)	(252)	(533)	(1330)	(804)
Cont	557	45	63	33	430	904	13	302	576	40	722	705	19	642	964	4482	11337	11382
	(83)	(4)	(5)	(11)	(65)	(84)	(9)	(51)	(119)	(32)	(158)	(70)	(11)	(71)	(152)	(614)	(1216)	(1106)
P=	0.75	1.00	0.82	0.584	0.94	0.84	0.84	0.15	1.00	0.22	0.69	0.84	0.50	1.00	0.31	0.32	0.31	0.44

Tr, *Trichophyton rubrum* conidia; PMA, phorbol 12-myristate 13-acetate; LPS, lypopolyssacharide; Bg, background or without stimulation; Cont, healthy donors

Data presented as mean (SE)
